# Effect of light conditions on trophic level and gene expression of partially mycoheterotrophic orchid, *Cymbidium goeringii*

**DOI:** 10.1080/15592324.2023.2180159

**Published:** 2023-02-19

**Authors:** Keita Chagi, Hiroaki Komoda, Masashi Murakami

**Affiliations:** a Graduate School of Science and Engineering, Chiba University, Chiba, Japan; b Graduate School of Science, Chiba University, Chiba, Japan

**Keywords:** Mycorrhizal association, plant responses to light, plant hormone signaling and regulation, plant fungal interactions, plant symbiont interactions

## Abstract

Partial mycoheterotrophic i.e., mixotrophic, plants are the species which partially depend on mycorrhizal fungi for its nutrients. Although some of these plants are known to show plasticity in the degree of fungal dependence induced by the changes in light condition, the genetic background of this plasticity is largely unsolved. Here, we investigated the relationships between environmental conditions and nutrient sources based on ^13^C and ^15^N enrichment in mixotrophic orchid *Cymbidium goeringii*. We also shaded them for 2 months and evaluated the effect of light condition on the nutrient sources based on the abundance of ^13^C and ^15^N and the gene expressions by RNA-seq based de novo assembly. The shading had no effect on isotope enrichment, possibly because of the translocation of carbon and nitrogen from the storage organs. Gene expression analysis showed the upregulation of genes involved in jasmonic acid response in leaves of the shaded plants, which suggests that the jasmonic acid played an important role in regulation of degree of dependence against the mycorrhizal fungi. Our results suggest that mixotrophic plants might be controlling their dependency against the mycorrhizal fungi by a common mechanism with the autotrophic plants.

## Introduction

Carbon (C) acquisition is one of the fundamental functions in plants. While most plants fixed the atmospheric CO_2_ through photosynthesis to fulfill their needs of C, several plants gained C through the mycorrhizal fungi mutualized with them. These plants are called mycoheterotrophic (MH) plants. The levels of dependency against mycorrhizas vary among plant species from fully mycoheterotrophic (FM) to partial mycoheterotrophic (mixotrophic; MX).^1–3^ FM plants are observed in a variety of plant lineages from liverworts to Angiosperms for about 880 extant species [^1^,b]. These FM species frequently lack the chlorophyll and both morphologically and ecologically differ from AT plants in regard to degenerated leaves and roots, adaptation to darker environments, or changes in pollination systems ^4–6^.

Although many plants have been found to be MH, the physiological mechanism and the genetic backgrounds of mycoheterotrophy are little known. Several studies have provided insight into the genetic backgrounds of the evolution of FM plants by comparing transcriptomes of AT and FM plants. Transcriptomics of two FM orchids suggested that the shift from autotrophy to mycoheterotrophy is associated with function losses rather than metabolic innovations.^7^ Another study focused on several monocots found convergent gene loss in FM plants across different lineages suggesting shared genetic backgrounds.^8^ The albinistic variants of MX plants, which lack chlorophyll, give perspective on the evolution of FM plants as they gain their carbon needs solely through mycorrhizal fungi, unlike normal (green) MX plants ^9–11^. Several studies comparing green and albinistic MX plants suggest that albinistic individuals show higher expression rate in gene related to nutrients transporting, suggesting those genes are playing important roles in nutrition exchange between plant and its mycorrhizal fungi ^11,12^. Some of these genes are also involved in mycorrhizal symbiosis in ancestral AT plants suggesting the diversion of the genetic system of AT plants to the MH plants ^11,13^.

On the other hand, some MX plants can also plastically change their dependency against mycorrhizal fungi according to their surrounding environments such as light availability.^14–18^ This plasticity may allow MX plants to live in various environments, from light open areas to dark habitats such as forest floor. Motomura *et al.*^19^ suggests that the dependency against mycorrhizal fungi of an MX plant *C. goeringii* varies among habitats, which might reflect the difference of light condition between the habitats. However, the gene expression between these habitats is not compared, and the habitats were distant so that the environment apart from light conditions might affect this result. Hence, the genetic mechanisms of adjusting dependency levels against mycorrhizal fungi in response to changes in the environmental conditions are still unclear. The phytohormones such as gibberellins, auxins, strigolactones and jasmonic acids are known to be the key factors controlling the mycorrhizal symbiosis in AT plants.^20–23^ Gibberellins and auxins control mycorrhizal colonization both positively and negatively through regulations of hyphal entry and arbuscule formation.^21,23,24^ Strigolactones promote the arbuscular mycorrhiza formation of *Lotus japonicus* and other plants^20^ and also initiate the interaction of non-arbuscular mycorrhizal (AM) fungi with an FM orchid.^25,26^ Jasmonic acids and its metabolite act throughout the communication between plants and fungi by inducing flavonoids that attract the mycorrhizas and directly affect the signaling cascade that controls the mycorrhizal symbiosis.^22^ Jasmonic acids are also known to control AM colonization according to the environmental light conditions.^27^ Mycorrhizal development is also affected by the environmental factors, such as availability of nutrients, water stresses, and light conditions, through the regulation of the phytohormones.^27–29^ Some of these phytohormones should also be involved in mycorrhizal symbiosis of MH plants by controlling the degree of mycoheterotrophy.^13^

*Cymbidium* is an orchid genus containing the MH species which varies in degree of mycoheterotrophy from AT (MH when juvenile) to almost FM ^19,30^. To name a few examples, *C. dayanum* is an AT plant with saprotrophic-endophytic rhizoctonias, while *C. goeringii* is an MX plant which has associations with both ectomycorrhizal fungi and saprotrophic-endophytic rhizoctonias.^31,32^ *Cymbidium macrorhizon* is an almost FM plant that has no leaves and photosynthesizes only on its scape and has associations only with ectomycorrhizal fungi.^30,31^ Therefore, *Cymbidium* can be a good model for testing the evolutionary process of MH plants phylogenetically, morphologically, and physiologically by comparing closely related species with different nutrient modes.

Here, we performed a field sampling and shading experiment on *C. goeringii* to test if the MX species, *C. goeringii*, is able to change their dependency against mycorrhizal fungi in response to the variation in light condition. Generally, the relative concentrations of ^13^C and ^15^N are enriched in MH plants by the nutrients derived from their mycorrhizal fungi.^34^ Although some studies pointed out that ^15^N concentrations are not directly affected by the light condition,^15,33,35,36^ we provisionally hypothesized that the shading interrupts the photosynthesis and induces higher dependence on fungi in shaded plants, resulting in higher ^13^C and ^15^N concentrations. We further compared the gene expression of shaded and unshaded individuals to evaluate the genes involved in the adjustment of the mycorrhizal symbiosis. Here we address the hypothesis that the mycorrhizal symbiosis in MX plants is, at least in part, regulated by the similar phytohormone-induced system with AT plants.

## Material and methods

### Sampling site and shading experiment

A population of *C. goeringii* was sampled at the temperate broadleaf deciduous forest located in the Ecological Garden of the Natural History Museum and Institute, Chiba (Chuo-ku, Chiba-Shi, Chiba-ken, Japan; E35.60°, N140.14°). For the first year of the observation (2018), we sampled 33 individuals of *C. goeringii* on 15th August and 24th October. For the second year of the observation (2019), we collected 32 individuals which are the same individuals as the last year (one individual was lost), and the additional 15 individuals on 15th August and 17th October. At the same time, we shaded 10 mature individuals of *C. goeringii* out of those 47 individuals by the frames covered with 60% black shade cloth (KLARK Co. Ltd., Japan). Each frame was covered with the cloth except for the bottom. The cloth was adjusted to be about 5–10 cm above the top of each plant. The shading was performed from 15th August 2019 until 17th October 2019 (64 d). The other 36 individuals were left unshaded and used as control.

### Measurement of environmental condition

We measured precipitation, temperature, and canopy openness as environmental conditions that might affect the dependency of *C. goeringii* against mycorrhizas. Precipitation and temperature were measured as the macro-environmental factors which might affect all individuals in the site equally, whereas canopy openness was measured as the micro-environmental factors which might affect each individual plant. The precipitation and temperature were measured by the Meteorological equipment of the Ecological Garden. The total precipitation and the average temperature over 10 d before the sampling date, respectively, were utilized (both values included the values on the sampling date). The photos of the canopy above each plant individual were taken by a built-in camera of a smartphone (AQUOS sense lite SH-M05; SHARP, Japan) equipped with a fisheye lens (Daiso, Japan), which were imported to CanopOn 2 (http://takenaka-akio.org/etc/canopon2/) to major canopy openness. To index the maturity of each individual of *C. goeringii*, the number of pseudobulbs was recorded. Because *C. goeringii* has a sympodial growth and makes new shoots (pseudobulbs) once a year, the number of the pseudobulbs reflects the plant age after shoot formation.

### Stable isotope analysis

About 1 cm of the leaf from the newest shoot of each individual of *C. goeringii* were collected seven times from 15th August to 17th October for the first year, and the first day (August, 15th) and the last day (October, 17th) of the shading for the second year. For the reference of δ^13^C and δ^15^N values, leaves (peduncles and rhizomes for the FM plant) of five AT (*Carpinus tschonoskii, Cleyera japonica, Machilus thunbergii, Pleioblastus chino, Quercus serrata*, and *Sasa nipponica*), one MX (*Ce. falcata*), and one FM (*C. macrorhizon*) plant species from the surrounding understorey were collected. The leaves were dried in a 60°C oven for 5 d and stored at room temperature until the weighing. Each 1.000 mg – 5.000 mg of the dried leaves were placed in a tin capsule, and δ^13^C and δ^15^N were measured at the UCDavis Stable Isotope Facility (U.S.A.). The relative abundance of C and N isotope ratios, i.e. δ^13^C and δ^15^N respectively, were calculated by the equation

$$\delta^{13}\;C\;or\;\delta^{15}N=\left(R_{sample}/R_{standard}-1\right)\times 1000[\%]$$

using Vienna Pee Dee Belemnite and atmospheric N_2_ as standards for C and N respectively, where R is the molar ratio of the isotopes, hence ^13^C/^12^C or ^15^N/^14^N. The standard deviations for replicate combustions of the internal standards were 0.05 ‰ for δ^13^C and 0.07 ‰ for δ^15^N respectively.

Differences in δ^13^C or δ^15^N among AT, MX, and FM plants were compared by Generalized linear model (GLM) adding nutrient modes as a fixed factor. We considered five models which contain all combinations of one to three groups of nutrient modes with null model. The best model was selected by Akaike’s information criterion (AIC) values. Simple regression analyses were used to determine relationships between each environmental factor and isotope ratios. The isotope data of shaded and unshaded individuals were compared with *glmer* function in the R package *lme4* to fit a generalized linear mixed model (GLMM) adding shading and sampling date as fixed factor and plant ID as a random factor.^37^ All statistical analyses were performed in R v4.1.2 [R ^38^].

### RNA extraction and RNA sequencing

About 3 cm of the leaf, 5 mm of the shoot tip (stem), and 1 cm of the matured root from the newest shoot, respectively, of each *C. goeringii* individual was collected and preserved in RNAlater (Invitrogen, U.S.A.) on the first day and the last day of the shading in the second year of the experiment (2019). The specimens were kept at −80°C until the total RNA extraction. Total RNA was extracted using Maxwell 16 LEV Plant RNA Kit with the Maxwell 16 Research Instrument (Promega, U.S.A.) according to the manufacturer’s instructions. RNA concentrations were measured using a Qubit 2.0 Fluorometer (Invitrogen, U.S.A.), and electrophoresis on agarose gel was performed to check the RNA degradation. RNA purity was estimated using a BioSpec-nano (Shimadzu, Japan). The cDNA library was constructed using TruSeq RNA Sample Prep Kits. Paired-end (150 bp) RNA sequencing (RNA-seq) was performed on the Illumina NovaSeq6000 platform.

### De novo assembly and detection of differentially expressed genes (DEGs)

We utilized Trimmomatic v.0.39 for the removal of adaptor sequences and low-quality reads,^39^ and used FastQC v.0.11.9 for quality control (http://www.bioinformatics.babraham.ac.uk/projects/fastqc/). The remaining reads were used for de novo assembly using Trinity v.2.13.2.^40^ To estimate gene expression levels, trimmed sequences of each sample were mapped to the reference transcripts using RSEM v.1.3.3.^41,42^ The read count data was used for gene expression analysis. The isoforms were concatenated by “Trinity_gene_splice_modeler.py” which is a part of the Trinity utilities, then we searched for homologues of every gene using BLAST searches for all protein sequences of *Arabidopsis thaliana* (Araport11). Genes with the best hit and with an e-value <0.0001 were used for gene expression analysis. DEGs among the two light conditions were detected using the DESeq2 package. We considered genes with q < 0.05 as DEGs. Gene ontology (GO) enrichment analysis of the DEGs was performed using the statistical enrichment test of the PANTHER classification system (http://pantherdb.org/).

## Results

### Stable isotope analysis

**Figure 1.**
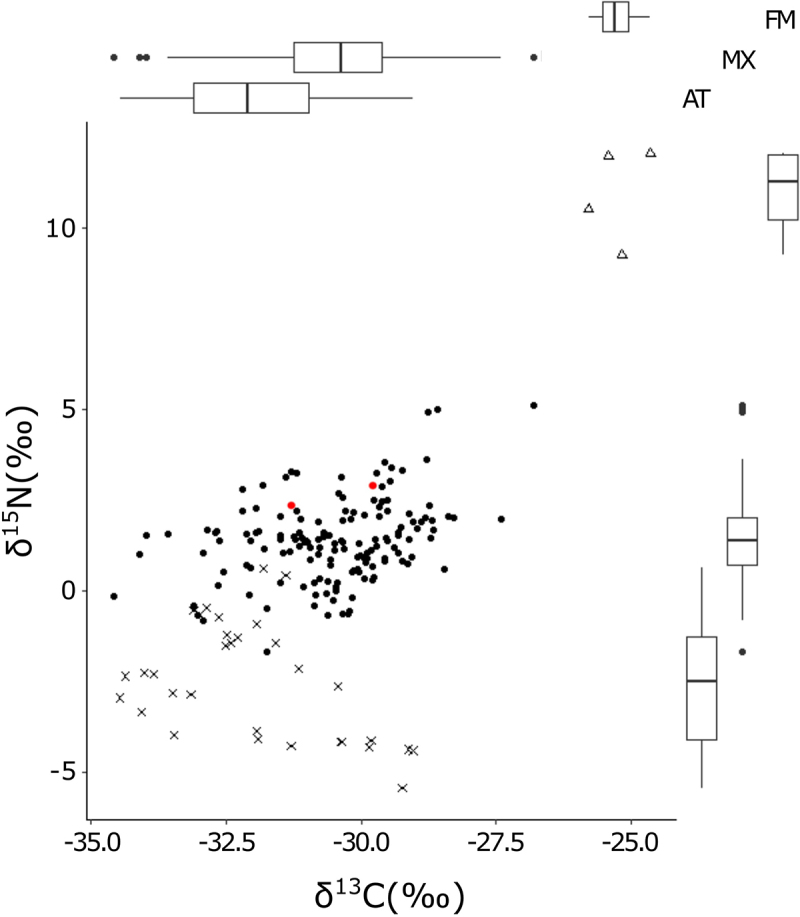
δ^31^C and δ^30^N values of autotrophic (AT; crosses), mixotrophic (MX; circles), and fully mycoheterotrophic (FM; triangles) plants. The box plots on the right side and the top represent δ^31^C values and δ^30^N of each trophic level, respectively. Two red circles indicate *Cephalanthera falcata*. See text and figure S1 for the included species.

**Figure 2.**
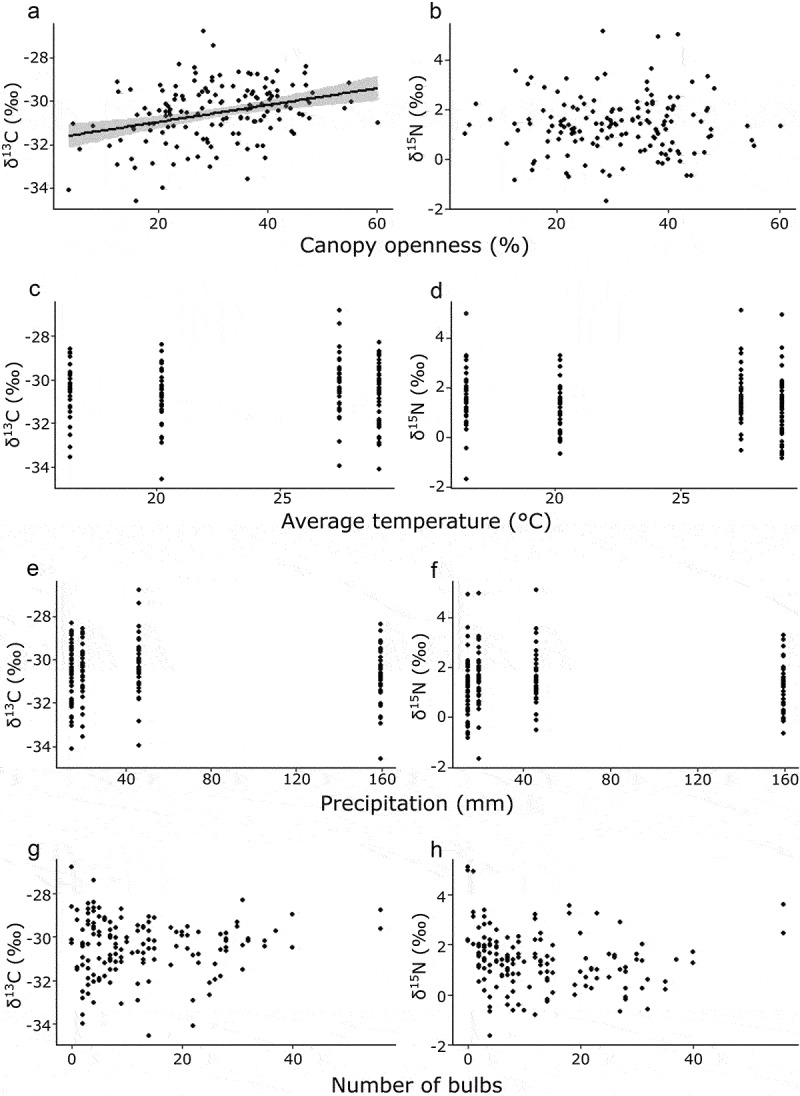
Correlation between the environmental factors and isotope ratios. Simple regression analysis was used to determinate each relationship. **A**, canopy openness and δ^31^C (*P* < .001); **B**, canopy openness and δ^30^N (*P* = .92); **C**, temperature and δ^31^C (*P* = .92), **D**; temperature and δ^30^N (*P* = .88); **E**, precipitation and δ^34^C (*P* = .57); **F**, precipitation and δ^30^N (*P* = .28); **G**, bulb numbers and δ^31^C (*P* = .53); and **H**, bulb numbers and δ^30^N (*P* = .097).

**Figure 3.**
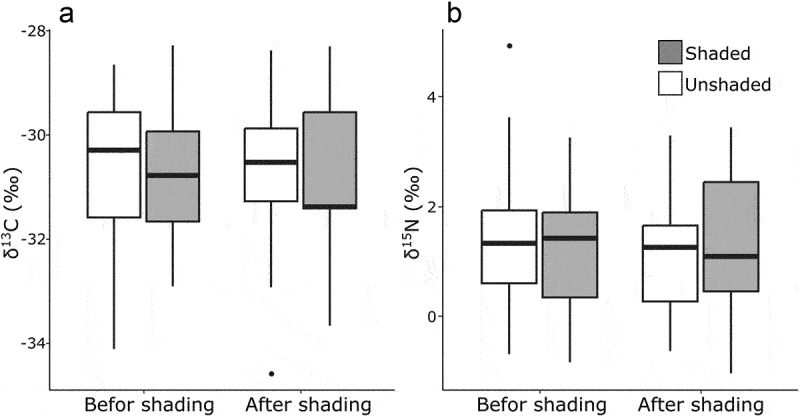
δ^31^C and δ^30^N values of leaf tissue in shaded and unshaded individuals of *C. goeringii*. The left plots are the values from the first day of the shading, and right plots are from the last day of the shading. Null models were selected based on AIC value. **A**, δ^31^C; **B**,δ^30^N.

The δ^13^C and δ^15^N values of the *C. goeringii* range widely, often higher than AT and lower than FM plants (Figure 1). The model that distinguishes three nutrient modes was selected based on AIC for both δ^13^C and δ^15^N values (AIC_best_ = 642, AIC_null_ = 716 for δ^13^C, AIC_best_ = 607, AIC_null_ = 856 for δ^15^N). The MX orchid species, *Ce. falcata*, showed the similar values with *C. goeringii* in both δ^13^C and δ^15^N. The δ^13^C values of unshaded *C. goeringii* ranged from −34.6‰ to −28.3‰ and showed a positive correlation with canopy openness (Figure 2a, p < .001; Tukey–Kramer test), while other environmental conditions i.e. temperature and precipitation and number of the bulbs have no effects on them (Figure 2c, e, g; p = .92, *P* = .57, *P* = .53, respectively). The δ^15^N values of unshaded *C. goeringii* ranged from −0.83‰ to −4.93‰. No effects of environmental conditions on the δ^15^N were detected (Figure 2b, d, f, h; canopy openness: *P* = .92; temperature: *P* = .88; precipitation: *P* = .28; number of bulbs: *P* = .10). The δ^13^C values of the shaded individuals ranged from −33.7‰ to −28.3‰, while the δ^15^N values ranged from −1.04‰ to −3.43‰ (Figure 3). When the δ^13^C and δ^15^N values of shaded and unshaded individuals were compared, none of the effects of shading were detected by the GLMM with the null model being selected based on AIC values.

### Difference in gene expressions under the shaded and unshaded conditions

Eighty-six genes were detected as DEGs including 73 upregulated and 13 downregulated ones expressed in the leaves of shaded individuals (Tables 1 and S1). Fourteen GO terms were detected as enriched for the “Biological process” category and all of them were over-expressed in the shaded individuals. There was a single GO related to jasmonic acid, the phytohormone related to mycorrhizal symbiosis (“response to jasmonic acid”, GO:0009753). Other GOs related to phytohormones, here gibberellins, auxins, and strigolactones, were not detected. One GO term from the “Cellular component” category was detected as enriched and was under-expressed in the shaded individuals (Table 2). No GO terms from the “Molecular function” category were enriched. Among the genes expressed in the stems, eight genes were identified as DEGs including three upregulated and five downregulated in shaded individuals (Tables 1 and S1). There were no genes related to phytohormones. No GO terms were detected as enriched for the genes expressed in the stems. For the genes expressed at the roots, none of them were detected as DEGs.

**Table 1.** Number of differential expressed genes found in each tissue of *Cymbidium goeringii.*

	upregulated	downregulated
Leaf	73	13
Stem	3	5
Root	0	0

**Table 2.** GO enrichment in leaf tissue of shaded individuals of *Cymbidium goeringii*. +: Upregulated GOs; -: Downregulated GOs.

GO Term (Accession No.)	Reference count	DEG count	Expression	FDR
**Biological process**				
Response to herbivore (GO:0080027)	7	3	+	1.53E-02
protein complex oligomerization (GO:0051259)	31	4	+	3.48E-02
Response to jasmonic acid (GO:0009753)	267	12	+	1.60E-03
Response to fatty acid (GO:0070542)	269	12	+	8.65E-04
Response to fungus (GO:0009620)	406	12	+	1.77E-02
Defense response to bacterium (GO:0042742)	416	12	+	1.91E-02
Response to bacterium (GO:0009617)	522	14	+	1.16E-02
Response to external biotic stimulus (GO:0043207)	1082	20	+	1.77E-02
Response to other organism (GO:0051707)	1082	20	+	1.58E-02
Response to biotic stimulus (GO:0009607)	1084	20	+	1.46E-02
Biological process involved in interspecies interaction between organisms (GO:0044419)	1092	20	+	1.47E-02
Defense response (GO:0006952)	988	18	+	3.40E-02
Response to external stimulus (GO:0009605)	1371	24	+	1.07E-02
Response to stress (GO:0006950)	2647	37	+	4.39E-03
**Cellular component**				
protein-containing complex (GO:0032991)	1977	1	-	1.38E-02
**Molecular function**				
No enriched GO terms				

## Discussion

### The effect of the environment against trophic levels of *C. goeringii*

The δ^13^C and δ^15^N values of *C. goeringii* showed a wider range than AT and FM plants (Figure 1). Motomura et al. (2010) showed that there are considerable differences in δ^13^C and δ^15^N values in *C. goeringii* inhabiting different localities, suggesting the ability to adjust the dependency against fungi in different macro-environments. Our results showed a further wider range of δ^13^C and δ^15^N values than those shown in Motomura *et al*. (2010) even among the individuals inhabiting the same locality. This suggests that this species has an ability to adjust the dependency against fungi not only under the different macro-environment but also under the different micro-environments.

We hypothesized that the individuals in darker places had higher ^13^C and ^15^N concentrations because of the higher C and N uptake from the mycorrhizal fungi. Our results showed that canopy openness had a positive effect on δ^13^C value and no effect on δ^15^N value, which is inconsistent with our hypothesis (Figure 2a-f). Apart from the mycorrhizal dependency, there are several factors that might affect the ^13^C and ^15^N concentrations, which are translocation, water stress, and soil conditions.^43–45^ Generally, the concentrations of ^13^C in leaves are increased when the water stress is high.^44^ The place with higher canopy openness might have higher irradiance which results in higher ground temperature and lower soil water content, which causes higher water stress resulting in higher ^13^C concentration. The other factor that might affect the ^13^C and ^15^N are translocation from the storage organs. Some studies suggested that the existing N pool affects δ^15^N values by diluting the newly acquired N from mycorrhizal fungi.^15,35^ *Cymbidium goeringii* is an evergreen perennial with pseudobulbs, thus δ^13^C and δ^15^N values in the new shoots might be affected by the isotope ratios of C and N stored in the pseudobulbs, which reflects the degree of mycoheterotrophy in the past few years, not only the present year.

The number of the pseudobulbs, i.e. plant maturity, showed any effect on the isotope ratios, although the individual with the highest δ^13^C and δ^15^N values was detected in the individual without any pseudobulbs (Figure 2g, h). After the germination, *C. goeringii* spends underground for several years gaining their nutritions from its mycorrhizal fungi, and produces vegetative shoot after that.^46^ Hence, the juvenile shoots may be produced mainly from the C and N from the fungi which show higher ^13^C and ^15^N concentrations. As the isotope ratios vary between the plants with one or more pseudobulbs, our results also suggest that the plants after producing the vegetative shoots quickly adjust their degree of mycoheterotrophy to the surrounding environments.

### The effect of the shading against the stable isotope ratio

Against our expectations, the shading had no effects on δ^13^C and δ^15^N values of *C. goeringii*. Since *C. goeringii* have evergreen, pseudobulbous shoots which last for several years,^46^ C and N stored in the old shoots may flow into the newer shoots. The isotope ratios of this “old” C and N might reflect in the result at the recent months, but years’ trends in degree of mycoheterotrophy of the individuals. In addition to the translocation, shading might also cause an impact on δ^13^C values via keeping the humidity inside the frame. Higher humidity reduces water stress, resulting in lower δ^13^C value,^44^ thus the increase in δ^13^C values due to the influence of mycorrhizal dependency may be counteracted by the influence.

### Difference in gene expressions among shaded and unshaded individuals

We could identify several DEGs between shaded and unshaded treatments on leaf and stem specimens, but not in root specimens (Table 1; S1). In the leaf tissues of shaded individuals, genes with the GO term “response to jasmonic acid” are over expressed. Jasmonic acid is one of the phytohormones synthesized in leaves^47^ and known to be one of the key factors that positively controls mycorrhizal colonization.^22^ In tomatoes and legumes, the level of jasmonic acid synthesis was affected by the density of the plant canopy ^48^, which enabled the control of AM colonization according to the environmental light conditions.^27^ Because major components of symbiosis of orchids with mycorrhizal fungi are known to be shared with AT plants with AM fungi,^13^ jasmonic acid in *C. goeringii* might also play a role in mycorrhizal control as for the AT plants. If this is the case, MX orchid may be controlling the dependency against the mycorrhizal fungi through the regulation of jasmonic acid synthesis by synthesizing them in a darker environment. Jasmonic acid is also known to induce the defensive reaction to the various biotic and non-biotic stresses.^49,50^ The multiples of the genes possibly induced by the jasmonic acid response were also enriched in the leaves under the shading, which are those with GO of “defence response”, “response to other organism”, “response to fungus”, “response to herbivore”, and “defence response to bacterium”. These reactions are congruent with the idea that there are conflicts between host plant and its mycorrhizal fungi, while the fungi might be pathogenic if their growth is excessive.^51^ However, jasmonic acid response can occur not only in mycorrhizal responses but also in responses to microorganisms other than mycorrhizal fungi and abiotic stresses.^49,50^ Thus, stresses related to the shading such as pathogens activity promoted by shading could also induce these genes. The GOs related to other phytohormones such as gibberellins, auxins and strigolactones were not found as over or under expressed. The synthesis of these phytohormones in the leaves might be not regulated by the light conditions, because they are often regulated in the root and affect mycorrhizal symbiosis.^20,21^ Additionally, GOs related to the transport of nutrients, such as sugars and amino acids, do not exhibit significant over or under expression. However, previous research on albinistic variants of MX plants has demonstrated that their leaves exhibit overexpression of genes involved in nutrient transport, likely as a compensatory mechanism to counteract C deficiency resulting from their inability to perform photosynthesis.^12^ This difference should be due to the fact that the shaded individuals may still produce sufficient C by photosynthesis and their severity of C shortage is not as high as to require degrading cellar components in the present experiment.

We found fewer DEGs in stem tissues compared to the leaves. C provided from the mycorrhiza may be supplied to the whole plant through stems, and the jasmonic acids are synthesized in the leaves and transported to the roots via vascular bundles.^52^ Although it is expected that the genes involved in the transporting process such as “Transport” and “Transporter activity” should respond to the increased C provision from roots and production of jasmonic acid in the leaves, we can not detect any DEGs in stem tissues. Several genes such as RNA-polymerase and elongation factor which are involved in the transcription process were detected as DEGs in stems (Table S1). Our samples included the shoot meristem, thus these genes might be affected by the shading and regulating the leaf formations to adapt to the darker environment.

For the root tissues, no DEGs were found between shaded and unshaded individuals, including phytohormone-related genes. The study on MX orchid species, *Epipactis helleborine*, showed that there are several DEGs which are related to symbiosis in roots between the green MX and albinistic FM individuals (Suetsugu et al., 2017). The mycorrhizal fungi of *E. helleborine* were restricted to *Wilcoxina* species group (at least for the study), while *C. goeringii* can symbiose with several groups of fungi such as saprotrophic-endophytic rhizoctonias and several ectomycorrhizal fungal taxa.^31,32^ In some cases, the same plant individuals are colonized by several species of the mycorrhizal fungi.^31^ We did not investigate the species composition of mycorrhizal fungi in *C. goeringii* in the present study. There might be a difference in composition of the fungi among each individual, and even between the roots of the same individual. If this is the case, the difference in above-ground environment might have less impact than the difference in mycorrhizal fungi composition in each part of the roots. This may explain the lack of DEGs between roots of shaded and unshaded individuals in *C. goeringii*.

## Conclusions

Although the stable isotope ratios of C and N had not changed during the shading experiment, changes in gene expressions suggest that the MX orchid *C. goeringii* changed their trophic levels in response to the light conditions of the surrounding environments. Our results indicate that the MX orchid *C. goeringii* might control their dependency against mycorrhizal fungi with jasmonic acid regulation in leaves, which could be a common mechanism with the AT plants. This suggests that MH plants adjust their dependency against fungi in response to changes in the environment as well as AT plants.

## Supplementary Material

Supplemental Material
