# Supplementary material for: Effect of light conditions on trophic level and gene expression of partially mycoheterotrophic orchid, Cymbidium goeringii
Source: Plant Signal Behav. 2023 Feb 19;18(1):2180159. doi: 10.1080/15592324.2023.2180159 (PMC9980479; doi:10.1080/15592324.2023.2180159)
Supplement: Supplemental Material [file KPSB_A_2180159_SM2996.docx]

Table S1. List of differential expressed genes found in each tissue of *Cymbidium goeringii*. +: overexpressed gene; -: underexpressed gene.

| Sequence ID | Expression | Best hit (BLAST search against *Arabidopsis* proteins) | Description (based on Araport 11) |
| --- | --- | --- | --- |
| **Leaf** |  |  |  |
| TRINITY_DN23135_c0_g1 | + | AT2G44800 | 2-oxoglutarate (2OG) and Fe(II)-dependent oxygenase superfamily protein |
| TRINITY_DN7279_c0_g1 | + | AT3G11180 | 2-oxoglutarate (2OG) and Fe(II)-dependent oxygenase superfamily protein |
| TRINITY_DN9705_c0_g1 | + | AT5G13490 | ADP/ATP carrier 2 |
| TRINITY_DN128_c1_g1 | + | AT5G16120 | alpha/beta-Hydrolases superfamily protein |
| TRINITY_DN3161_c0_g2 | + | AT5G37710 | alpha/beta-Hydrolases superfamily protein |
| TRINITY_DN6606_c1_g1 | + | AT5G37710 | alpha/beta-Hydrolases superfamily protein |
| TRINITY_DN19712_c0_g1 | + | AT5G53050 | alpha/beta-Hydrolases superfamily protein |
| TRINITY_DN32488_c0_g1 | + | AT1G02660 | alpha/beta-Hydrolases superfamily protein |
| TRINITY_DN12862_c0_g1 | + | AT3G10960 | AZA-guanine resistant1 |
| TRINITY_DN27386_c0_g1 | - | AT1G62710 | beta vacuolar processing enzyme |
| TRINITY_DN5169_c0_g1 | + | AT2G24240 | BTB/POZ domain with WD40/YVTN repeat-like protein |
| TRINITY_DN11464_c0_g1 | + | AT5G05240 | cation-transporting ATPase |
| TRINITY_DN11923_c0_g1 | - | AT5G13930 | Chalcone and stilbene synthase family protein |
| TRINITY_DN5838_c0_g1 | - | AT5G13930 | Chalcone and stilbene synthase family protein |
| TRINITY_DN39192_c1_g1 | - | AT3G22840 | Chlorophyll A-B binding family protein |
| TRINITY_DN4828_c2_g1 | - | AT4G37970 | cinnamyl alcohol dehydrogenase 6 |
| TRINITY_DN878_c0_g1 | - | AT5G43310 | COP1-interacting protein-like protein |
| TRINITY_DN4933_c0_g1 | + | AT4G24460 | CRT (chloroquine-resistance transporter)-like transporter 2 |
| TRINITY_DN1121_c0_g1 | + | AT2G36130 | Cyclophilin-like peptidyl-prolyl cis-trans isomerase family protein |
| TRINITY_DN1287_c8_g1 | - | AT4G23160 | cysteine-rich RECEPTOR-like kinase |
| TRINITY_DN33119_c0_g1 | + | AT3G01900 | cytochrome P450 |
| TRINITY_DN4375_c0_g1 | - | AT2G41120 | DUF309 domain protein |
| TRINITY_DN772_c2_g1 | + | AT2G38410 | ENTH/VHS/GAT family protein |
| TRINITY_DN23374_c0_g1 | + | AT3G23240 | ethylene response factor 1 |
| TRINITY_DN16988_c0_g1 | + | AT4G35930 | F-box family protein |
| TRINITY_DN24344_c0_g1 | + | AT1G70140 | formin 8 |
| TRINITY_DN7249_c0_g1 | - | AT4G32940 | gamma vacuolar processing enzyme |
| TRINITY_DN7611_c0_g1 | + | AT1G78440 | gibberellin 2-beta-dioxygenase |
| TRINITY_DN9070_c0_g1 | + | AT3G62760 | Glutathione S-transferase family protein |
| TRINITY_DN5559_c1_g1 | + | AT5G32470 | heme oxygenase-like |
| TRINITY_DN7506_c0_g1 | + | AT4G37680 | heptahelical protein 4 |
| TRINITY_DN1747_c0_g1 | + | AT4G21870 | HSP20-like chaperones superfamily protein |
| TRINITY_DN14523_c0_g1 | + | AT5G01210 | HXXXD-type acyl-transferase family protein |
| TRINITY_DN2123_c6_g3 | + | AT4G11430 | hydroxyproline-rich glycoprotein family protein |
| TRINITY_DN159_c0_g1 | + | AT1G76070 | hypothetical protein |
| TRINITY_DN7893_c0_g1 | + | AT3G23230 | Integrase-type DNA-binding superfamily protein |
| TRINITY_DN571_c0_g1 | + | AT5G64750 | Integrase-type DNA-binding superfamily protein |
| TRINITY_DN18410_c0_g1 | + | AT3G21720 | isocitrate lyase |
| TRINITY_DN8370_c0_g1 | + | AT3G45140 | lipoxygenase 2 |
| TRINITY_DN3520_c1_g1 | + | AT4G34950 | Major facilitator superfamily protein |
| TRINITY_DN25857_c0_g2 | + | AT2G45040 | Matrixin family protein |
| TRINITY_DN2424_c0_g2 | + | AT1G64660 | methionine gamma-lyase |
| TRINITY_DN1986_c0_g1 | + | AT5G55090 | mitogen-activated protein kinase kinase kinase 15 |
| TRINITY_DN3642_c1_g1 | + | AT1G49920 | MuDR family transposase |
| TRINITY_DN7538_c0_g1 | - | AT3G13080 | multidrug resistance-associated protein 3 |
| TRINITY_DN7349_c0_g1 | + | AT3G13540 | myb domain protein 5 |
| TRINITY_DN7349_c0_g2 | + | AT3G13540 | myb domain protein 5 |
| TRINITY_DN2166_c0_g1 | + | AT1G69850 | nitrate transporter 1:2 |
| TRINITY_DN5066_c1_g1 | + | AT3G20660 | organic cation/carnitine transporter4 |
| TRINITY_DN648_c0_g1 | + | AT4G11650 | osmotin 34 |
| TRINITY_DN1788_c1_g1 | + | AT1G78230 | Outer arm dynein light chain 1 protein |
| TRINITY_DN2528_c0_g1 | + | AT5G48840 | pantoate-beta-alanine ligase |
| TRINITY_DN1485_c3_g2 | + | AT1G78780 | pathogenesis-related family protein |
| TRINITY_DN17817_c0_g1 | + | AT5G24070 | Peroxidase superfamily protein |
| TRINITY_DN4086_c0_g2 | + | AT1G52200 | PLAC8 family protein |
| TRINITY_DN63210_c0_g1 | + | AT2G35930 | plant U-box 23 |
| TRINITY_DN6371_c0_g1 | + | AT2G35930 | plant U-box 23 |
| TRINITY_DN2090_c0_g1 | - | AT3G61870 | plant/protein |
| TRINITY_DN6765_c0_g1 | - | AT1G33970 | P-loop containing nucleoside triphosphate hydrolases superfamily protein |
| TRINITY_DN7130_c0_g1 | + | AT1G76600 | poly polymerase |
| TRINITY_DN20614_c0_g1 | + | AT1G28390 | Protein kinase superfamily protein |
| TRINITY_DN4865_c0_g1 | + | AT1G54820 | Protein kinase superfamily protein |
| TRINITY_DN70959_c0_g2 | + | AT1G07160 | Protein phosphatase 2C family protein |
| TRINITY_DN2263_c0_g1 | + | AT1G55230 | proteinase inhibitor I4 |
| TRINITY_DN3134_c0_g2 | + | AT4G14680 | Pseudouridine synthase/archaeosine transglycosylase-like family protein |
| TRINITY_DN4784_c0_g1 | + | AT5G21930 | P-type ATPase of Arabidopsis 2 |
| TRINITY_DN12136_c0_g1 | + | AT5G50400 | purple acid phosphatase 27 |
| TRINITY_DN2130_c1_g1 | + | AT1G34060 | Pyridoxal phosphate (PLP)-dependent transferases superfamily protein |
| TRINITY_DN3337_c1_g1 | + | AT3G15060 | RAB GTPase homolog A1G |
| TRINITY_DN4723_c0_g1 | - | AT4G21470 | riboflavin kinase/FMN hydrolase |
| TRINITY_DN19539_c0_g1 | + | AT5G20885 | RING/U-box superfamily protein |
| TRINITY_DN2272_c0_g2 | + | AT3G24255 | RNA-directed DNA polymerase (reverse transcriptase)-related family protein |
| TRINITY_DN1524_c0_g1 | + | AT2G41380 | S-adenosyl-L-methionine-dependent methyltransferases superfamily protein |
| TRINITY_DN1046_c0_g1 | + | AT1G32740 | SBP (S-ribonuclease binding protein) family protein |
| TRINITY_DN4289_c0_g1 | + | AT4G10170 | SNARE-like superfamily protein |
| TRINITY_DN4801_c2_g1 | + | AT4G32480 | sugar phosphate exchanger |
| TRINITY_DN13033_c4_g1 | + | AT4G16740 | terpene synthase 03 |
| TRINITY_DN331_c1_g1 | + | AT1G61120 | terpene synthase 04 |
| TRINITY_DN591_c3_g1 | + | AT1G74950 | TIFY domain/Divergent CCT motif family protein |
| TRINITY_DN4787_c0_g2 | + | AT2G36750 | UDP-glucosyl transferase 73C1 |
| TRINITY_DN562_c0_g1 | + | AT2G36760 | UDP-glucosyl transferase 73C2 |
| TRINITY_DN2116_c2_g2 | + | AT1G68450 | VQ motif-containing protein |
| TRINITY_DN5231_c1_g1 | + | AT1G80840 | WRKY DNA-binding protein 40 |
| TRINITY_DN1953_c0_g1 | + | AT5G64810 | WRKY DNA-binding protein 51 |
| TRINITY_DN378_c3_g1 | + | AT5G13080 | WRKY DNA-binding protein 75 |
| TRINITY_DN9795_c0_g1 | + | AT5G13080 | WRKY DNA-binding protein 75 |
| **Stem** |  |  |  |
| TRINITY_DN20977_c0_g2 | - | AT2G25010 | Aminotransferase-like plant mobile domain family protein |
| TRINITY_DN38558_c0_g1 | + | AT5G12110 | elongation factor 1-beta 1 |
| TRINITY_DN6710_c0_g1 | - | AT1G50610 | Leucine-rich repeat protein kinase family protein |
| TRINITY_DN1044_c0_g1 | + | AT3G59220 | pirin |
| TRINITY_DN1903_c0_g1 | + | AT3G49500 | RNA-dependent RNA polymerase 6 |
| TRINITY_DN15967_c0_g2 | - | AT5G51080 | RNase H family protein |
| TRINITY_DN19086_c0_g1 | - | AT4G37925 | subunit NDH-M of NAD(P) H:plastoquinone dehydrogenase complex |
| TRINITY_DN877_c0_g1 | - | AT4G13980 | winged-helix DNA-binding transcription factor family protein |
